# A single-center retrospective study of dabrafenib plus trametinib combination therapy in patients with *BRAF V600E*-positive thyroid cancer

**DOI:** 10.1007/s10147-025-02861-8

**Published:** 2025-08-22

**Authors:** Taiju Ando, Yuko Oya, Yumi Tomiie, Kimio Ogawa, Tomoki Kuki, Yosuke Tanabe, Hisayuki Kato, Kazuyoshi Imaizumi, Yatsuka Hibi, Kenji Kawada

**Affiliations:** 1 Department of Medical Oncology, Fujita Health University, 1-98 Dengakugakubo, Kutsukake-cho, Toyoake, Aichi 470-1192 Japan; 2 Department of Respiratory Medicine, Fujita Health University, Toyoake, Aichi Japan; 3 Department of Endocrine Surgery, Fujita Health University, Toyoake, Aichi Japan; 4 Department of Otolaryngology-Head and Neck Surgery, Fujita Health University, Toyoake, Aichi Japan

**Keywords:** BRAF V600E, Thyroid cancer, Dabrafenib, Trametinib

## Abstract

**Background:**

*BRAF V600E* mutation is a key oncogenic driver commonly found in papillary thyroid carcinoma (PTC) and anaplastic thyroid carcinoma (ATC). Dabrafenib plus trametinib combination therapy targeting this mutation has shown promising activity in clinical trials. This study aimed to verify the real-world effectiveness of this combination therapy in patients with *BRAF V600E*-positive PTC and ATC.

**Methods:**

We retrospectively investigated adult patients with *BRAF V600E*-positive PTC and ATC who were treated with dabrafenib plus trametinib at a single university hospital in Japan.

**Results:**

Between January 2024 and December 2024, 10 patients with PTC and 6 patients with ATC were identified, among whom 13 patients (81%) had been previously treated with lenvatinib. An objective response was observed in 5 patients with PTC (50%) and 4 patients with ATC (67%). The median progression-free survival and overall survival were not reached in patients with PTC, and were 2.7 months and 3.6 months, respectively, in patients with ATC. Partial regression of intracranial metastatic tumors was observed in 1 of the 2 patients with brain metastases at baseline. Rechallenge with another BRAF/MEK inhibitor combination therapy, encorafenib plus binimetinib, was attempted in 2 patients with ATC and demonstrated clinically meaningful responses.

**Conclusions:**

Dabrafenib plus trametinib combination therapy demonstrated clinical effectiveness in patients with *BRAF V600E*-positive thyroid cancer, with response rates comparable to those observed in clinical trials. However, tumor shrinkage did not appear to translate into improved survival in patients with ATC, highlighting the limitations of current targeted therapies in managing this aggressive subtype.

## Introduction

Thyroid cancer includes various histological subtypes with distinct clinical characteristics. Papillary thyroid carcinoma (PTC) is a major subtype of differentiated thyroid carcinoma and generally has favorable outcomes. In contrast, anaplastic thyroid carcinoma (ATC) is rare (~2% of thyroid malignancies) but is highly aggressive and difficult to control, even with multimodal treatment. Additionally, a small subset of PTC undergoes anaplastic transformation and is fatal [1]. The *BRAF V600E* mutation, which is identified in approximately 80% of PTCs and 50% of ATCs in Japanese patients with thyroid cancer, drives tumorigenesis *via* MAPK pathway activation [2, 3]. Targeted therapies that combine BRAF and MEK inhibitors have shown promising tumor-agnostic clinical activity against *BRAF V600E*-positive solid tumors. In Japan, dabrafenib plus trametinib combination therapy was approved for *BRAF V600E*-positive solid tumors, including thyroid cancers, in November 2023, and another combination therapy, encorafenib plus binimetinib, was approved for *BRAF V600E*-positive thyroid cancers in May 2024 [4, 5]. However, given the substantial differences in the clinical and biological features among thyroid cancer subtypes, assessing the efficacy of BRAF/MEK inhibitor treatments in each subtype in real-world settings is crucial to improve the treatment strategies and define the role of tumor-agnostic therapies in *BRAF V600E*-positive thyroid cancer. This study aimed to verify the real-world clinical efficacy and safety of dabrafenib plus trametinib combination therapy in Japan.

## Materials and methods

### Study design and patient selection

This retrospective study analyzed data from patients who received treatment at Fujita Health University Hospital, a regional core hospital with 1376 beds designated as a Cancer Genome Medicine Cooperation Hospital and a Designated Cancer Care Hospital, between January 2024 and December 2024. This study included all eligible patients ≥ 18 years of age with histologically confirmed *BRAF V600E*-positive PTC or ATC who initiated dabrafenib plus trametinib combination therapy during the study period, without applying any specific exclusion criteria. Patients with pathologically confirmed anaplastic transformation of PTC were included in the analysis.

Patients received combination therapy with oral dabrafenib (150 mg, twice daily) plus oral trametinib (2 mg, once daily) until disease progression, unacceptable toxicity, patient withdrawal, or death, with dose reductions implemented as needed. Computed tomography, usually performed within 4 weeks before treatment initiation, was reviewed as a baseline evaluation. Imaging studies to assess the response to treatment were typically performed regularly every 2 months or as clinically indicated. The response to treatment was assessed based on the Response Evaluation Criteria in Solid Tumors (RECIST) version 1.1, and adverse events (AEs) were graded according to the Common Terminology Criteria for Adverse Events (CTCAE) version 5.0. Progression-free survival (PFS) and overall survival (OS) were calculated from the date on which treatment with dabrafenib plus trametinib was initiated, and patients who remained on treatment at the data cut-off point were censored at that time.

### Statistical analysis

All statistical analyses were performed using R (version 4.4.1; R Foundation for Statistical Computing, Vienna, Austria) and R Studio (version 2024.12.0+467). Survival analyses were conducted using the survival package (version 3.7-0), and data visualization was performed using ggplot2 (version 3.5.1). The cut-off date was December 31, 2024. Owing to the limited sample size, no subgroup analyses were performed. Survival outcomes were estimated using the Kaplan–Meier method. Descriptive statistics were reported as medians and ranges for continuous variables, and frequencies and percentages for categorical variables.

## Results

### Patients

The study cohort included 16 patients, consisting of 10 patients with PTC and six with ATC (Table 1). Four patients in the ATC group underwent anaplastic transformation of PTC. *BRAF V600E* mutation was confirmed in tumor specimens from all patients using the MEBGEN^TM^ BRAF 3 kit (Medical & Biological Laboratories Co., Ltd., Japan), which employs polymerase chain reaction with sequence-specific oligonucleotide probes (PCR-rSSO).

**Table 1.**
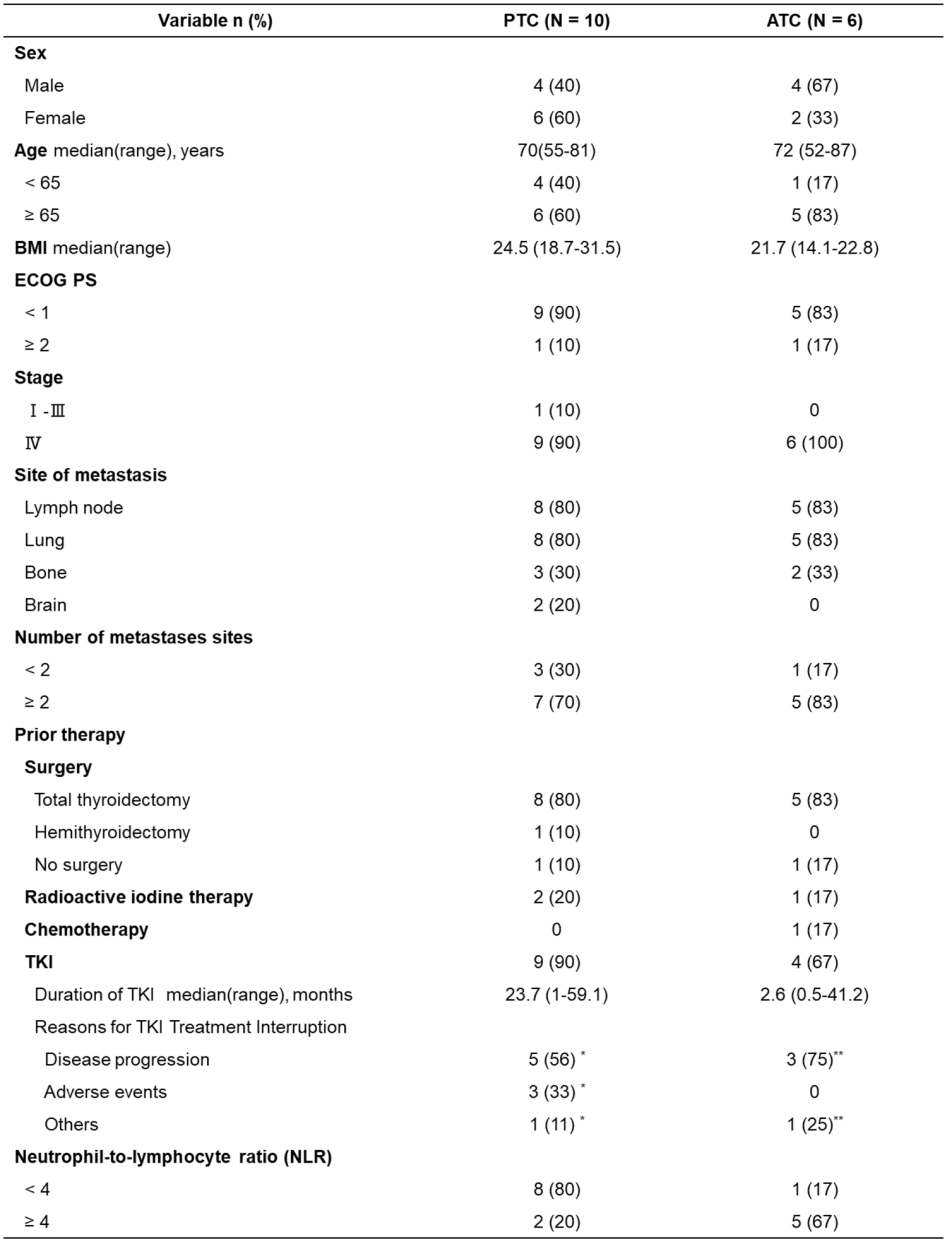
Baseline characteristics

*ECOG PS* Eastern Cooperative Oncology Group Performance Status, *TKI* tyrosine kinase inhibitor

^*^Percentages are based on *N* = 9. **Percentages are based on *N* = 4

All patients had documented disease progression within 12 months before the initiation of dabrafenib plus trametinib combination therapy. Two patients with PTC had brain metastases, both of whom had previously undergone stereotactic radiotherapy for their lesions. One patient with ATC received paclitaxel monotherapy. Nine patients with PTC (90%) and 4 with ATC (67%) were treated with lenvatinib, a multi-tyrosine kinase inhibitor (TKI). The median duration of TKI treatment was 23.7 months (range 1–59.1) and 2.6 months (range: 0.5–41.2) for patients with PTC and ATC, respectively. Reasons for prior TKI discontinuation included disease progression, adverse events, and other factors such as the interruption of treatment for surgery. One patient with PTC did not receive TKI treatment because of concerns regarding the risk of bleeding. The other two patients began treatment after BRAF/MEK inhibitors were approved in Japan. Since their approval, our institution has adopted a policy of using BRAF/MEK inhibitor combination therapy as first-line treatment for patients with BRAF V600E-positive ATC. In two patients with ATC, treatment was rechallenged with another BRAF/MEK inhibitor combination therapy: encorafenib plus binimetinib.

### Treatment efficacy

**Fig. 1:**
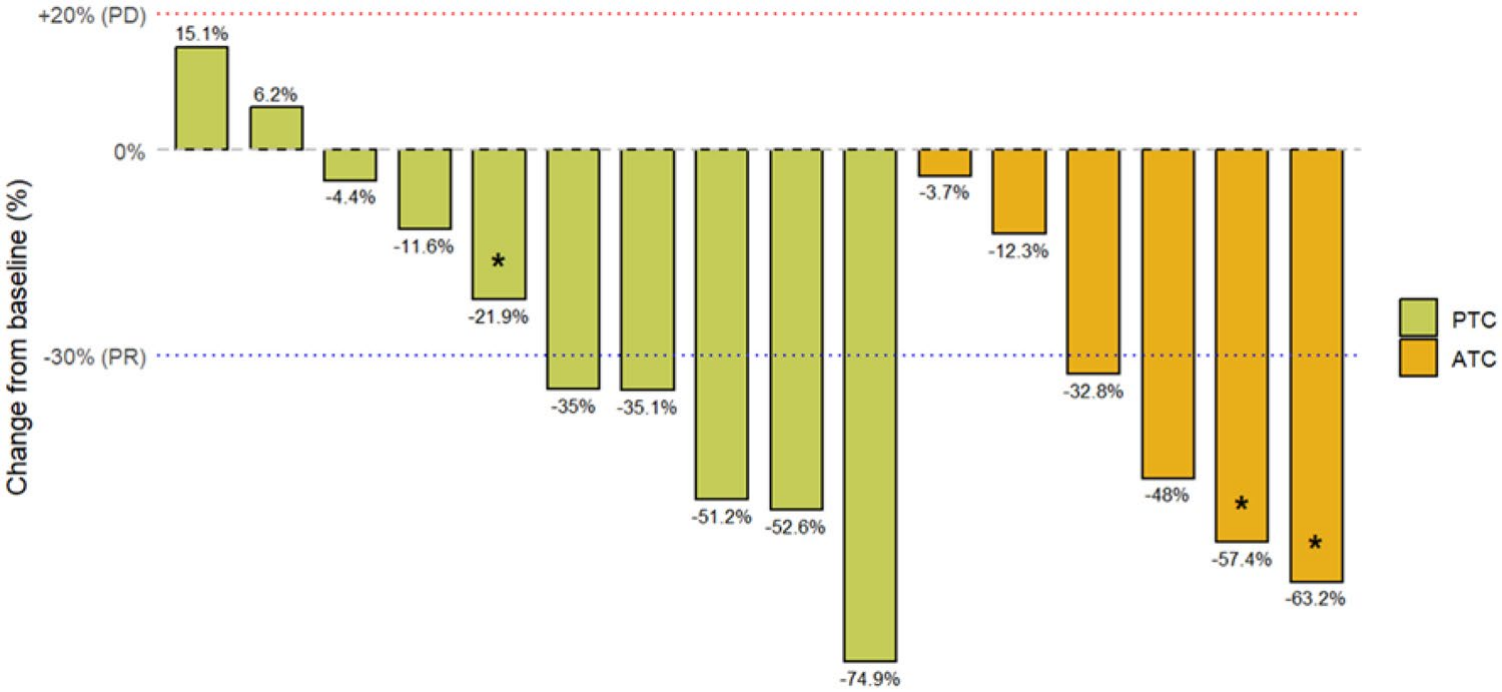
Waterfall plot of best percentage change in tumor size from baseline. The best percent change from baseline in the sum of the longest diameters for extracranial target lesions ranged from –74.9 to + 15.1% in patients with PTC and from – 63.2 to – 3.7% in patients with ATC. Asterisks (*) indicate patients who received the dabrafenib plus trametinib combination as the first-line therapy

At the data cut-off point, 7 patients with PTC remained on treatment, while all 6 patients with ATC either discontinued treatment or died. In 3 patients (1 with PTC and 2 with ATC) who had difficulty with oral intake, medications were administered *via* a nasogastric tube using a simple suspension method. The best percentage change from baseline in the sum of the longest diameters for extracranial target lesions ranged from – 74.9 to + 15.1% in patients with PTC and from – 63.2 to – 3.7% in patients with ATC (Fig. 1). An apparent objective response (> 30% decrease in the sum of the longest diameters of the target lesion) was observed in 5 patients with PTC (50%, 95% CI 24–76%) and 4 patients with ATC (67%, 95% CI 24–94%). There were no cases with complete disappearance of the tumor. Stable disease was observed in the remaining 5 patients with PTC (50%) and 2 patients with ATC (33%). None of the patients showed progressive disease as the best response.

**Table 2.**
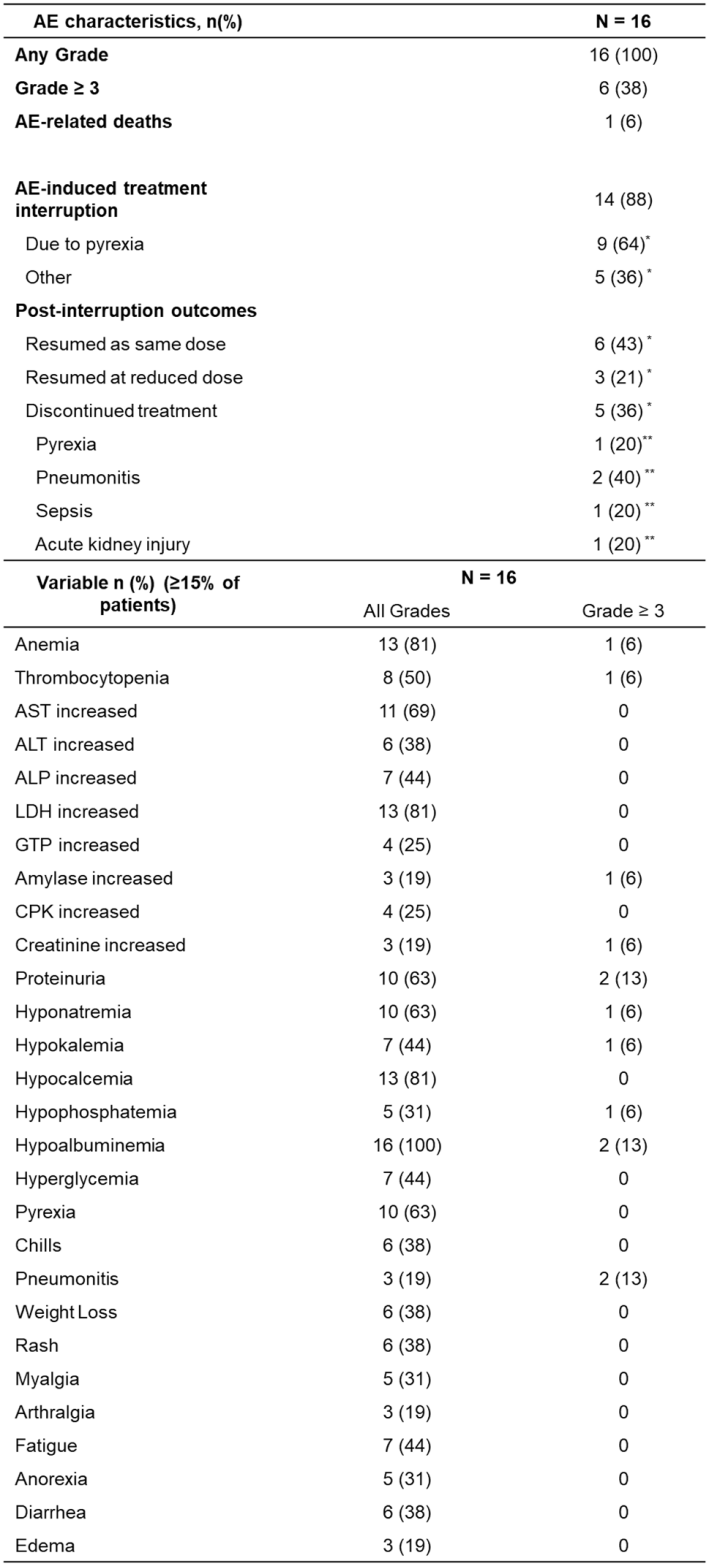
Summary of adverse events and treatment outcomes

*AE* adverse events.

^*^Percentages are based on *N* = 14. **Percentages are based on *N* = 5

**Fig. 2:**
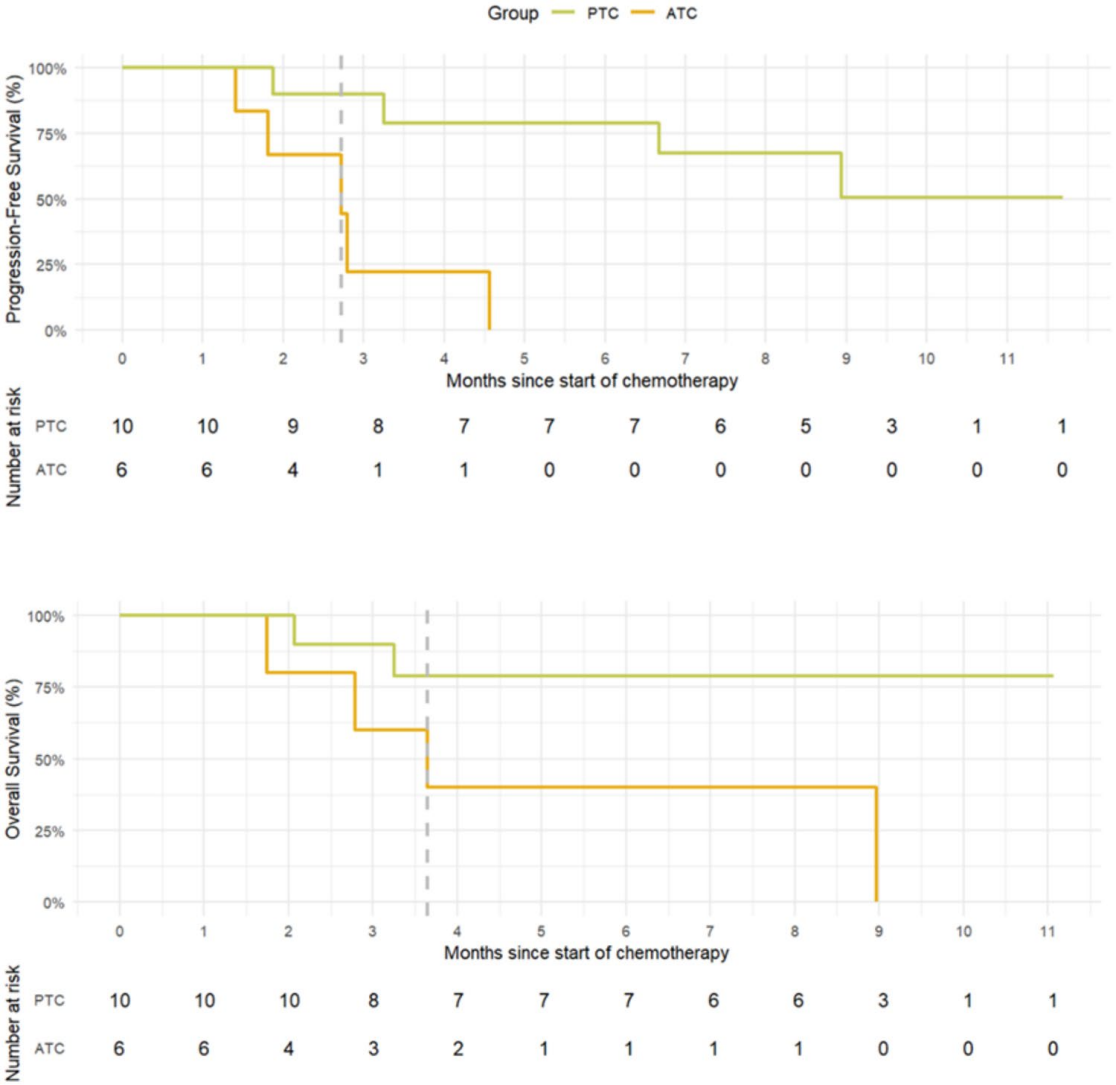
Kaplan–Meier curves for progression-free survival and overall survival. Kaplan–Meier curves for PFS (upper) and OS (lower) with the number of patients at risk. The median PFS was not reached in patients with PTC and was 2.7 months in patients with ATC. The median OS was not reached in patients with PTC and was 3.6 months in patients with ATC. PFS, progression-free survival; OS, overall survival

**Fig. 3:**
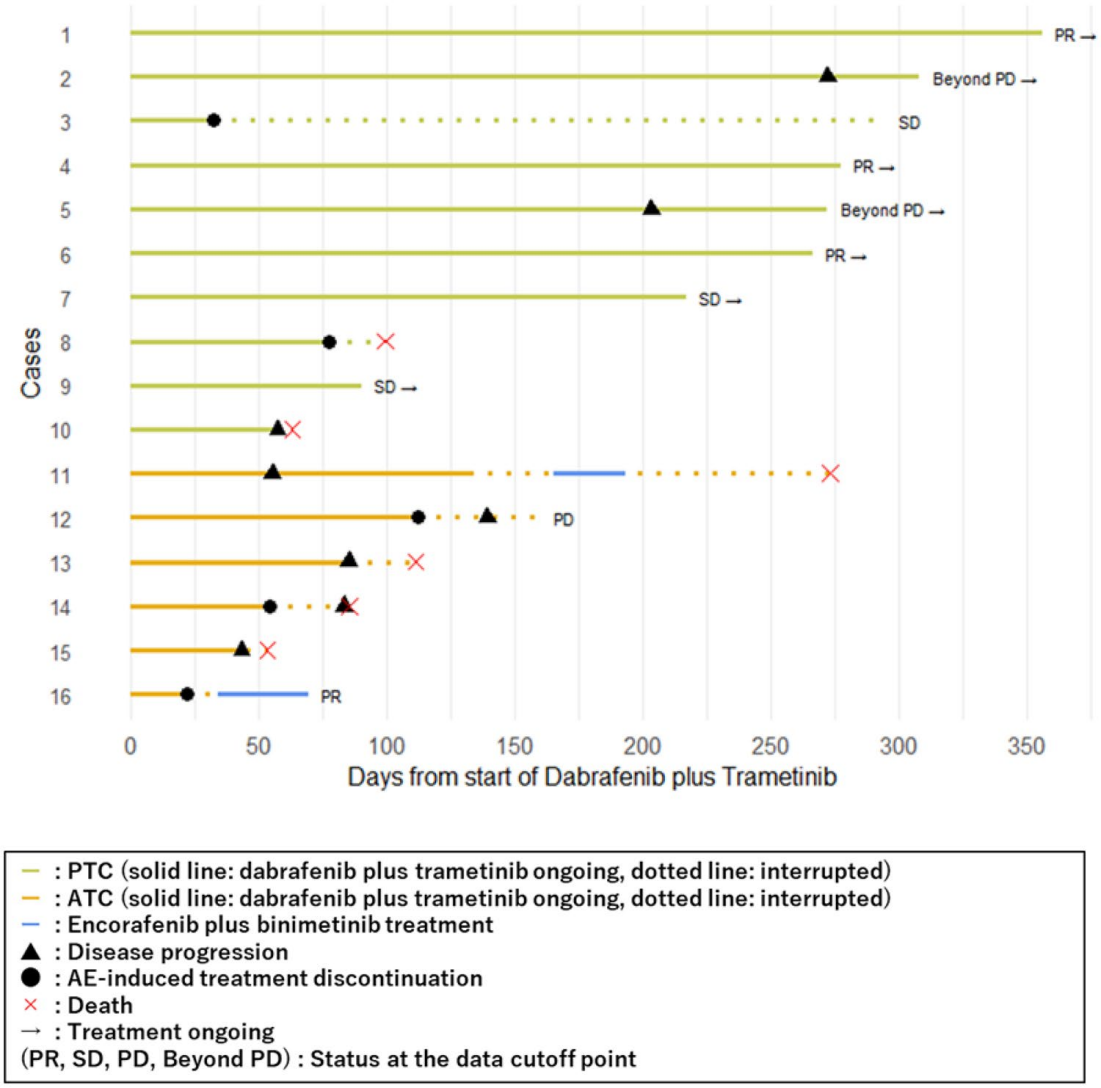
Swimmer plot of treatment duration and outcomes. The swimmer plot summarizes the treatment duration and outcomes for each patient. The treatment response indicated on the right (PR, SD, PD, and Beyond PD) represents the status at the data cut-off and does not reflect the best response. Cases 7, 14, and 16 received dabrafenib plus trametinib combination as the first-line therapy, while all other patients had prior treatment with lenvatinib. PR, partial response; SD, stable disease; PD, progressive disease

The median follow-up time was 8.8 months (range 2.1–11.7) for patients with PTC and 3.2 months (range 1.7–9.0) for patients with ATC. The median PFS was not reached in patients with PTC and 2.7 months (95% CI 1.8–not estimable [NE]) in patients with ATC (Fig. 2). The median OS was not reached in patients with PTC, whereas it was 3.6 months in patients with ATC (95% CI2.8–NE, Fig. 2). Figure 3 shows a swimmer plot summarizing the treatment duration and outcomes for each patient. Patients with PTC tended to have a longer treatment duration than those with ATC.

Of the 2 PTC patients with brain metastases at baseline, 1 achieved a partial response, with a 39.7% reduction in intracranial disease and a 74.9% reduction in extracranial disease. The patient was a 64 year-old woman who developed multiple brain metastases (diameter < 3 cm) in the cerebrum, midbrain, and cerebellum. Before the initiation of dabrafenib plus trametinib combination therapy on day 1, stereotactic radiotherapy was administered to 10 intracranial lesions between days − 57 and − 43 (25–35 Gy/5 fractions). However, on day − 12, progression of the brain, lung, and cervical lymph-node metastases was observed. Tumor shrinkage was confirmed in both intracranial and extracranial lesions on day 34 and was sustained until the data cut-off point. In the other patient with brain metastases, the intracranial response could not be evaluated until the data cut-off point. Additionally, a patient with ATC who did not have intracranial disease at baseline developed diplopia on day 50 of treatment, and imaging demonstrated a new midbrain metastasis. Despite concurrent stereotactic radiotherapy and continuing dabrafenib plus trametinib combination therapy, the patient died of disease progression before symptomatic improvement.

### Sequential BRAF/MEK inhibitor rechallenge

The first case was a 52 year-old woman (Case 11 in Fig. 3) who had been treated with lenvatinib previously. The patient experienced 48% tumor reduction with dabrafenib (300 mg daily) plus trametinib (2 mg daily) combination therapy before tumor progression was observed on day 55. The treatment was continued until day 133 because of potential clinical benefits without any dose reduction. Subsequent carboplatin plus paclitaxel combination chemotherapy was ineffective. Treatment with another BRAF/MEK inhibitor combination therapy, encorafenib (450 mg daily) plus binimetinib (90 mg daily), was initiated on day 165. On day 167, the patient developed grade 2 serous retinal detachment, necessitating a dose reduction to encorafenib (300 mg daily) and binimetinib (60 mg daily). Despite transient shrinkage of some lymph nodes, the treatment was discontinued, because new metastases were observed on day 193. The patient died due to disease progression on day 274.

The second case involved a 67 year-old woman (Case 16 in Figure 3) who initially achieved 57% tumor reduction with dabrafenib (300 mg daily) plus trametinib (2 mg daily). However, treatment was discontinued on day 23 because of treatment-related grade 3 acute kidney injury. Following renal function recovery, encorafenib (450 mg daily) plus binimetinib (90 mg daily) were readministered on day 34. The patient’s grade 3 creatinine level increased on day 38, requiring immediate treatment interruption. After recovery, treatment was resumed on day 49, with dose reduction of encorafenib (300 mg daily) and binimetinib (60 mg daily). Tumor shrinkage was maintained until day 140, with no further recurrence of renal dysfunction.

### Safety

All 16 patients experienced at least one AE, with 6 patients (38%) experiencing grade 3 or worse AEs, including drug-induced pneumonitis, proteinuria, and hypoalbuminemia in 2 patients (13%) (Table 2). Pyrexia was observed in 10 patients (63%), most of whom had grade 1 disease. The median time to the onset of pyrexia was 6.5 days (range 1–48 days), and the median time to recovery was 1 day (range 1–5, Table3). Eye disorders, possibly caused by trametinib, were limited to a single case of grade 1 severity. One treatment-related death was documented during the study period due to drug-induced interstitial lung disease. The patient was an 82 year-old woman with PTC and no prior history of pulmonary disease. Multiple pulmonary and bilateral cervical lymph-node metastases were observed, and dabrafenib plus trametinib combination therapy was initiated on day 1. The treatment was initially well tolerated; however, on day 80, pyrexia (> 38°C) developed, and the therapy was discontinued. CT revealed bilateral ground-glass opacities consistent with interstitial lung disease. The patient was hospitalized and treated with methylprednisolone (80 mg/day, 1 mg/kg) along with empirical antibiotics. The patient’s respiratory condition temporarily improved, and the steroid dose was tapered to 40 mg/day on day 93. Nevertheless, pyrexia and respiratory deterioration recurred on day 99. Repeat CT showed worsening interstitial changes, warranting the initiation of high-dose methylprednisolone (1000 mg/day). Despite treatment, the patient died of respiratory failure on day 100. Bronchoscopy was not performed because of the patient’s advanced age and poor performance status. All cultures were negative and the clinical course was consistent with drug-induced interstitial lung disease associated with dabrafenib plus trametinib therapy.

**Table 3.**
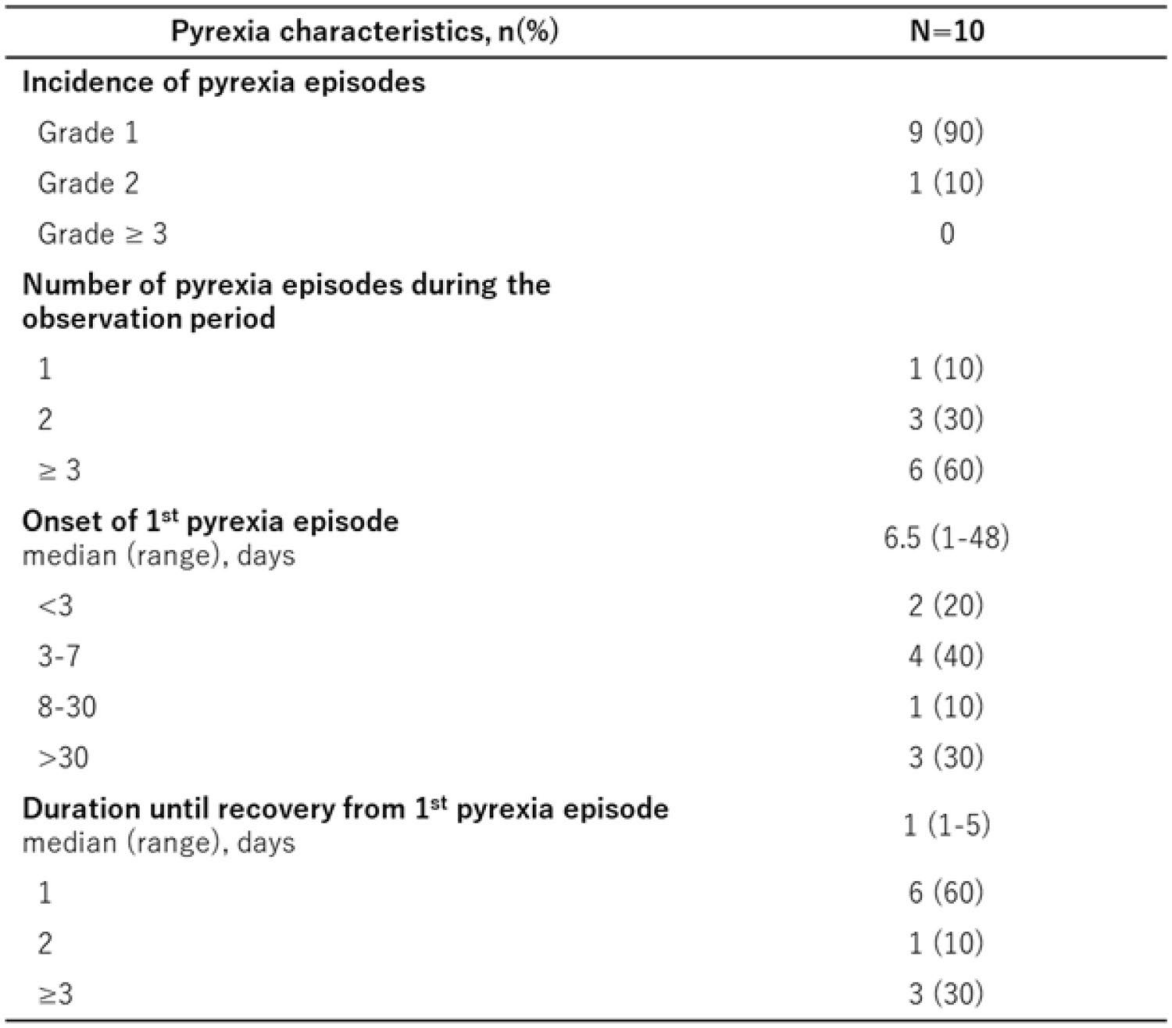
Characteristics of pyrexia episodes

Fourteen patients (88%) required at least one treatment interruption due to AEs (pyrexia in 9 cases). Only one patient discontinued treatment due to pyrexia.

## Discussion

This study describes the real-world efficacy and safety of dabrafenib and trametinib combination therapy in patients with *BRAF V600E*-positive thyroid cancer in Japan. The objective response rate of 50% in patients with PTC in this study was comparable to that in a previous phase 2 trial (30% or 48%, including minor responses [6]). Another retrospective study of dabrafenib plus trametinib combination therapy in *BRAF V600E*-positive thyroid cancer reported an objective response rate of 73.1% in 27 patients, and the high response rate in that study may have been due to the high proportion of early-stage cases [7]. The objective response rate of 67% in patients with ATC in this study was also comparable to that of the ATC cohort from the prior basket phase 2 trial (56%) and other retrospective analyses (50–100%) [4, 8–13]. Interestingly, in this study, the response rate in patients with ATC was higher than that in patients with PTC. A similar trend was reported in a phase 2 trial of encorafenib plus binimetinib combination therapy conducted in Japan; the objective response rates in patients with differentiated thyroid cancer and anaplastic thyroid cancer were 47.1% (8 of 17 patients) and 80.0% (4 of 5 patients), respectively [5]. Although bias due to the small number of patients with ATC cannot be excluded, intratumoral heterogeneity in *BRAF V600E*-positive PTC may have contributed to the poor response rate [14]. More importantly, tumor shrinkage did not appear to translate into improved survival in ATC patients. Previous reports indicate that for ATC patients ≥ 45 years of age, the median survival ranges from 1.6 to 4.8 months, even with multimodal therapy incorporating surgery, radiotherapy, and chemotherapy [15]. Despite the limited follow-up period and patient demographics, such as older age and the higher proportion of patients with advanced-stage disease in this cohort, the short PFS and OS in patients with ATC suggest that treatment with dabrafenib plus trametinib combination therapy may only provide a temporary benefit against this extremely aggressive subtype in real-world settings.

In recent years, tumor-agnostic approaches have gained attention, representing a paradigm shift from tissue-based to genomics-driven treatment decisions in medical oncology. However, as these tumor-agnostic basket trials focused on response rates, careful interpretation is needed across cancer types with distinct biological behaviors. For example, the selective tropomyosin receptor kinase (TRK) inhibitor larotrectinib demonstrated high efficacy in patients with NTRK fusion-positive PTC and follicular thyroid carcinoma (ORR 86%, 12 month PFS 100%, *n*=22), but it was less effective in treating those with ATC (ORR 29%, 12 month PFS 17%, *n*=7) [16]. *RET* and *ALK* fusions or high microsatellite instability are potential targets in thyroid cancer. However, given the possible variability in efficacy across each thyroid cancer subtype, particularly in patients with ATC, the prioritization of multi-TKI treatments and tumor-agnostic therapies thus requires careful evaluation.

One of the two patients with brain metastases at baseline obtained partial regression of intracranial metastatic tumors, which verified the intracranial activity of this combination therapy, although the response may have been influenced by prior radiotherapy. Another patient with brain metastases also showed a 48.6% reduction in intracranial lesions on imaging performed after the data cut-off point. Similar intracranial efficacy has been demonstrated in melanoma in a phase 2 trial [17]. Therefore, this combination therapy may also be effective against central nervous system lesions in thyroid cancer.

Another BRAF/MEK inhibitor combination, encorafenib plus binimetinib, was rechallenged in two patients with ATC, and both patients demonstrated clinically meaningful responses. As suggested in patients with melanoma [18], these findings indicate that the two treatments are not completely cross-resistant, which is important information for patients who have few treatment options or hypersensitivity to either drug.

Grade ≥3 AEs, including fatal interstitial pneumonia in 1 patient, occurred in 6 patients (38%). This rate was apparently lower in comparison to prior clinical trials (48-58%) [6, 19]. It is likely that effective management strategies derived from previous clinical trials, such as the identification of risk factors and monitoring methods, will be effectively utilized in real-world settings. Pyrexia, which often leads to treatment interruption, was successfully managed with dose modifications and the administration of antipyretics. Considering the unfavorable patient characteristics in this study, the rates of treatment interruption and discontinuation due to adverse events were considered to be within acceptable ranges, although real-world data may not fully capture the comprehensive spectrum of AEs.

The present study was associated with several limitations. As this was a single-center retrospective study, potential selection and assessment biases may exist. The overall adverse event profile may have been affected by the inclusion of patients with prior TKI treatment. The accumulation of further data is required to establish the appropriateness of prioritizing tumor-agnostic therapies for *BRAF V600E*-positive thyroid cancer.

## Conclusion

This study demonstrated that dabrafenib plus trametinib is effective in Japanese patients with *BRAF V600E*-positive thyroid cancer, with ORRs of 50% for PTC and 67% for ATC. However, despite initial tumor reduction in ATC, long-term disease control was limited. Adverse events were common but were generally manageable, although one treatment-related death occurred. These findings highlight the need for further research on optimized treatment strategies to improve survival outcomes.

## Data Availability

The data analyzed during this study are not publicly available due to patient privacy but are available from the corresponding author upon reasonable request and with institutional approval.
